# Trafficking of Hepatitis C Virus Core Protein during Virus Particle Assembly

**DOI:** 10.1371/journal.ppat.1002302

**Published:** 2011-10-20

**Authors:** Natalie A. Counihan, Stephen M. Rawlinson, Brett D. Lindenbach

**Affiliations:** Section of Microbial Pathogenesis, Yale University School of Medicine, New Haven, Connecticut, United States of America; University of California, San Diego, United States of America

## Abstract

Hepatitis C virus (HCV) core protein is directed to the surface of lipid droplets (LD), a step that is essential for infectious virus production. However, the process by which core is recruited from LD into nascent virus particles is not well understood. To investigate the kinetics of core trafficking, we developed methods to image functional core protein in live, virus-producing cells. During the peak of virus assembly, core formed polarized caps on large, immotile LDs, adjacent to putative sites of assembly. In addition, LD-independent, motile puncta of core were found to traffic along microtubules. Importantly, core was recruited from LDs into these puncta, and interaction between the viral NS2 and NS3-4A proteins was essential for this recruitment process. These data reveal new aspects of core trafficking and identify a novel role for viral nonstructural proteins in virus particle assembly.

## Author Summary

Hepatitis C virus (HCV) infects almost 200 million people worldwide, causing both acute and chronic liver disease. Although some antiviral treatments exist, they are not fully effective against all HCV genotypes and have serious side effects. In order to develop more effective treatment strategies, a better understanding of how HCV replicates in infected cells is required. In our study, we developed methods to visualize early steps in HCV particle assembly by fluorescently labeling core protein, a structural component of the virus. Soon after protein translation, core trafficked to the surface of large, immobile lipid droplets that were adjacent to sites of virus assembly. Core was also observed in highly motile puncta that traveled along microtubules. By using inhibitors of virus assembly and assembly-deficient viral mutants, we showed that core is recruited from lipid droplets into these puncta, and that this process was mediated by the interaction of HCV nonstructural proteins. Our work describes new methods to study the trafficking of core protein in infected cells, allowing us to better define aspects of infectious HCV particle assembly.

## Introduction

**Figure 1:**
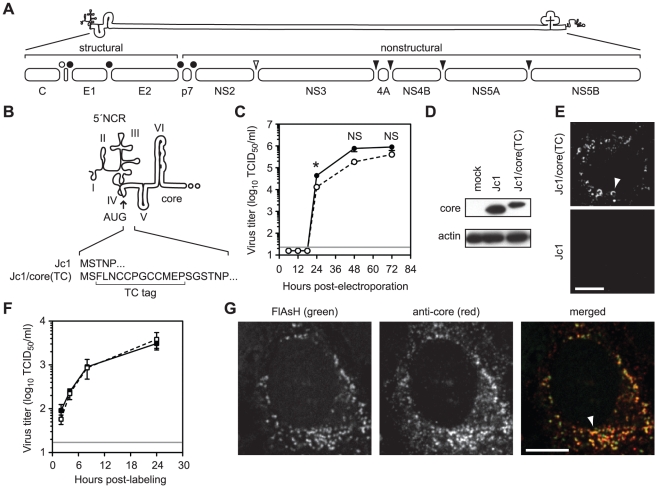
Fluorescent labeling of functional HCV core protein. (A) Schematic of HCV genome and polyprotein. The open bullet represents a signal peptide peptidase cleavage site; closed bullets represent signal peptidase cleavage sites; the open arrowhead represents the NS2–3 cysteine autoprotease cleavage site; closed arrowheads represent NS3-4A serine protease cleavage sites. (B) Schematic of TC tag inserted into the C terminus of core to create Jc1/core(TC). (C) Time course of Jc1 (closed circle) and Jc1/core(TC) (open circle) virus production after RNA electroporation. Values are averages of results from three independent transfections ± SEM. The grey line shows the limit of detection of this assay; the asterisk denotes the timepoint at which infectivity titers were significantly different (Student's unpaired t-test, p <0.05); NS denotes timepoints where the differences in infectivity titers were not statistically significant (p >0.05). (D) Western blot analysis of core protein expression from cells lysed at 48 h post-electroporation with the indicated viral transcript. Actin is shown as a loading control. (E) Live cell imaging of core protein. Huh-7.5 cells were infected with Jc1 or Jc1/core(TC) and labeled with FlAsH at 48 h post-infection. TC-tagged core was frequently observed in crescent structures (top panel, arrowhead) and in small puncta. Background signals were minimal with untagged Jc1 (bottom panel). (F) Effect of FlAsH labeling on virus assembly. Huh-7.5 cells were infected with Jc1/core(TC) and labeled with FlAsH or a mock label at 48 h post-infection. Supernatants were collected at 2, 4, 8, and 24 h post-labeling and titrated for infectivity. Values are averages from three replicates ± SEM. The grey line shows the limit of detection of this assay. No statistically significant difference (unpaired Student's t-test, p >0.05) was observed between FlAsH and mock-labeled cells at any time point. (G) Specificity of biarsenical labeling for core protein. Huh-7.5 cells were infected with Jc1 or Jc1/core(TC), labeled with FlAsH at 48 h post-infection, then fixed and stained for IF with anti-core antibody. Co-localization was observed in the merged image. For all micrographs, scale bars represent 10 µm.

Hepatitis C virus (HCV) is a major cause of acute and chronic hepatitis, cirrhosis, and hepatocellular carcinoma. HCV is an enveloped, positive-strand RNA virus classified with the Family *Flaviviridae* [1]. The viral genome encodes an open reading frame of ≈3011 codons that is translated as a single polyprotein, which is cleaved by viral and host proteases into at least 10 distinct products (Figure 1A). The N-terminal region encodes three structural components: core protein, which forms the viral nucleocapsid, and two envelope glycoproteins (E1 and E2), which mediate viral attachment and entry. The remainder of the genome encodes the nonstructural (NS) proteins: p7, NS2, NS3, NS4A, NS4B, NS5A and NS5B. The NS proteins mediate intracellular aspects of the virus life cycle including RNA replication, subversion of innate antiviral defense, and virus particle assembly. The precise roles of NS proteins in virus particle assembly are not clear but p7, NS2, NS3, NS4A, NS4B, and NS5A all contribute to this process [2], [3], [4].

HCV core is a highly basic RNA-binding protein that contains three distinct functional domains [5]. Domain 1 (amino acid (aa) 1–117) is hydrophilic and contains determinants for RNA binding and core oligomerization [6]. Domain 2 (aa 118–177) forms a pair of amphipathic helices that mediate the peripheral association of core with cellular membranes [7], [8], [9]. Domain 3 (aa 178–191), which serves as a signal peptide for the translocation of E1 protein into the endoplasmic reticulum (ER) lumen, is absent from mature core protein [5]. Core is initially cleaved from the polyprotein by host signal peptidase (SP); subsequent removal of domain 3 by signal peptide peptidase (SPP) then yields mature core protein that forms a homodimer [6], [9], [10].

Following cleavage, mature core protein is targeted to lipid droplets (LDs) [11], [12], [13]. LDs are intracellular storage organelles containing a hydrophobic core of neutral lipids and cholesterol esters surrounded by a phospholipid monolayer embedded with LD-specific proteins [14]. LD biogenesis is not fully understood, but LDs are likely derived from the outer leaflet of the ER and may remain contiguous with this membrane system [15]. LD-associated proteins are presumably loaded onto LDs at sites of ER contact [16], although vesicular transport mechanisms have not been formally excluded. The best-characterized LD-associated proteins are perilipin, adipocyte differentiation-related protein (ADRP), and tail-interacting protein (TIP) 47, collectively known as the PAT proteins [17]. PAT proteins are thought to regulate the dynamics of lipid acquisition, storage, and release [15]. In addition, the membrane trafficking GTPase Rab18 may associate with a subset of LDs undergoing lipolysis [18], [19], [20], [21].

The role of core trafficking to LDs is not well understood. Prior work has shown that core protein recruits NS proteins and RNA replication complexes to sites adjacent to LDs [22]. Furthermore, core recruits NS5A to the surface of LDs, where they co-localize [23]. Mutations that alter the LD localization of core or that block core's ability to recruit viral NS proteins to LDs inhibit virus production [22], [24], [25], [26], suggesting that LDs are intimately involved in virus particle assembly. The site of virus budding has not been definitively determined, but the ER retention of E1-E2 [27], [28], the complex glycan modifications on secreted virus particles [29], the differential effects of Brefeldin A (BFA) on virus assembly vs. virus secretion [30], and analogies to closely related flaviviruses (reviewed in [31]), all suggest that virus particles bud into the ER and transit through the secretory pathway. However, it is not yet clear how LD-associated core contributes to this process.

We hypothesize that core protein must be trafficked from LDs into nascent virus particles at the LD-ER interface. To understand the dynamics of core protein trafficking during virus assembly, we developed methods to fluorescently label and image functional core protein in live, virus producing cells. We observed core trafficking to static ADRP-positive LDs, forming a cap on the surface. At these sites, core co-localized with the viral E2 glycoprotein and adjacent to NS3 protein, consistent with these being sites of virus assembly. We also observed highly motile ADRP-independent core puncta that represent post-LD form of core. By using pharmacologic inhibitors of virus egress and a panel of mutants blocked in virus assembly, we showed that core is recruited from sites of assembly into these puncta, and that this process requires interaction between viral NS proteins.

## Results

### Fluorescent labeling of functional HCV core protein

To better understand the trafficking of HCV core protein during virus particle assembly, we developed methods to fluorescently label and image functional core in living cells. Traditional live imaging systems often rely on the insertion of fluorescent proteins into a target protein, but the relatively large size of such tags would likely interfere with the function of core protein. We therefore genetically inserted the small tetracysteine (TC) peptide tag (FLNCCPGCCMEP) near the N-terminus of core (Figure 1B) within the context of the HCV Jc1 infectious clone. Importantly, insertion of this tag had only minimal effects on infectious virus production compared to untagged Jc1 (Figure 1C). For both untagged Jc1 and Jc1/core(TC), peak viral infectivity was observed between 48 and 72 h post-electroporation, although the infectivity titers of Jc1/core(TC) were slightly reduced (2- to 4-fold) at each time point. This correlated with a 2.7-fold decrease in specific infectivity (0.16 Jc1 infectious units per RNA-containing particle vs. 0.06 Jc1/core(TC) infectious units per RNA-containing particle), suggesting that the small decrease in infectivity titers may have been due to an inefficiency in virus entry rather than in virus assembly. Both Jc1 and Jc1/core(TC) had similar biophysical profiles, with peak infectivities and specific infectivities observed in fractions with buoyant density of ≈1.10 g/ml (Figures S1A and S1B). Furthermore, the TC-tagged form of core accumulated to similar levels as untagged core within virus-producing cells (Figure 1D), and the TC insertion was retained after six serial virus passages (data not shown). Together, these data indicated that the TC tag insertion was well tolerated and that the TC-tagged core protein was functional for virus assembly.

To label core(TC), infected Huh-7.5 cells were incubated with FlAsH (green) or ReAsH (red) under optimized labeling conditions (as described in Materials and Methods) during the peak of virus assembly, 48 to 72 h post-infection or electroporation (Figure 1C). As shown in Figure 1E, specific signals were observed in Jc1/core(TC)-infected cells, often as bright puncta or crescents (Figure 1E, arrowhead), but not in Jc1-infected cells. Importantly, these FlAsH labeling conditions had no effect on the release of infectious virus particles (Figure 1F), indicating that these methods allowed us to label functional core protein during virus assembly. To further confirm that FlAsH-labeling was specific for core, Jc1/core(TC)-infected cells were labeled with FlAsH, fixed, and stained for core by IF (Figure 1G). FlAsH and anti-core IF signal largely co-localized (Pearson's correlation coefficient = 0.715), confirming that FlAsH labeled core(TC) protein. However co-localization was incomplete, largely due to the increased signal intensity of the IF signal in a perinuclear, reticular pattern. This difference could be attributable to one or more of the following issues. First, FlAsH signal intensity decreased 4-fold during fixation and processing for IF (Figure S2), mostly likely because this dye lacks an aldehyde-reactive primary amine and may be washed out during processing. Second, indirect IF is designed to amplify weak signals, whereas FlAsH labeling binds stoichiometrically to the TC tag. Third, FlAsH is less photostable than the Alexa dye used during IF, which could bias the relative signal intensities. Fourth, small dyes and antibodies may differ in their accessibility and efficiency of protein labeling. Nevertheless, these data confirmed that the TC tag can be used to label core protein in live, virus-producing cells.

### Localization and kinetics of core protein in live cells

To better clarify the role of core LD-trafficking during virus assembly, we created Huh-7.5-derived cell lines that stably expressed ADRP, a marker of storage LDs, fused to green fluorescent protein (GFP) or cerulean fluorescent protein (CFP). As ADRP expression levels can influence LD metabolism [32], we first characterized these cell lines for their ADRP expression, LD content, and ability to support infectious HCV particle assembly. Neither tagged-ADRP protein was overexpressed when compared to endogenous levels of ADRP expression (Figure S3C). There was a modest increase in the number of LDs in Huh-7.5/CFP-ADRP cells (170.8±67.89 LDs/cell) compared to Huh-7.5′s (128.0±53.1 LDs/cell), although this difference was not statistically significant (p >0.05; unpaired Student's t-test). Furthermore, there was no difference in the volume of LDs, either with or without oleic acid supplementation (Figure S3D). Importantly, both cell lines supported infectious virus production at levels comparable to non-transduced cells (Figure S3A–B). These data indicated that the GFP-ADRP and CFP-ADRP cells provided suitable environments to study core-LD trafficking in live cells.

**Figure 2:**
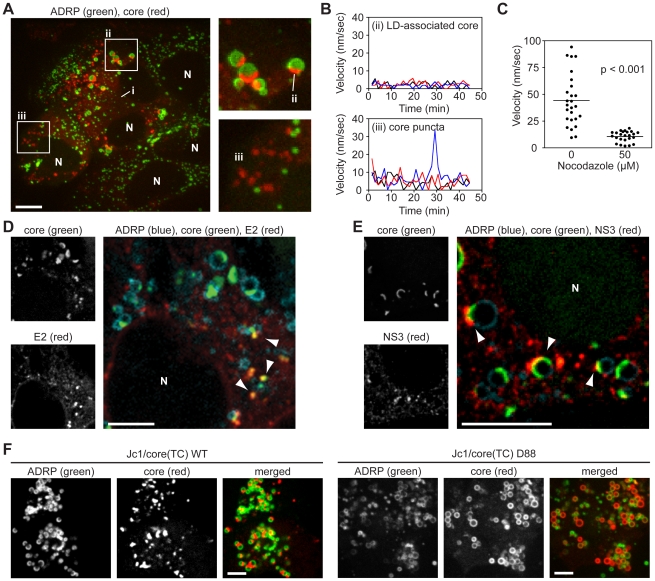
Localization and kinetics of core protein in live cells. (A) Localization of core in Huh-7.5 cells. Cells expressing GFP-ADRP were labeled with ReAsH at 48 h post-infection. Three forms of core were observed; i) ER-associated core; ii) core associated with ADRP-positive LDs; (iii) ADRP-independent core puncta. Panels show a magnification of ADRP-associated core as a cap on LDs (ii), and ADRP-independent core puncta (iii). Contrast was enhanced to show ER-associated core. Nuclei (N) are marked for reference. (B) Kinetics of intracellular core protein movement. The velocities of three representative LD-associated core particles (from Figure 2Aii) and core puncta (from Figure 2Aiii) were determined over the course of 45 minutes (0.75 frame/min) and the velocity at each timepoint shown. Individual particles were tracked by using Volocity Quantitation software. (C) Core puncta are sensitive to treatment with Nocodazole. Cells expressing GFP-ADRP were labeled with ReAsH at 48 h post-infection, then treated with 50 µM Nocodazole for 1 h. The velocity of individual ADRP-independent core particles in treated and untreated samples (n = 26) was determined over 10 frames (1 frame/min) by using Volocity Quantitation software. Values show average velocity of each particle over 10 frames, horizontal bars represent mean values of each group. The mean velocity of Nocodazole-treated cells was significantly different to non-treated cells (unpaired Student's t-test). (D–E) Co-localization of core with E2 and NS3. Cells expressing ADRP-CFP were labeled with FlAsH at 48 h post-infection, then fixed and stained for IF with anti-E2 (D) or anti-NS3 (E) antibodies. Nuclei (N) are shown for reference in the merged images. (D) Puncta of core co-localized with E2 puncta in areas adjacent to LDs (arrowheads). (E) NS3 localized to LDs containing caps of core (arrowheads). (F) Localization of core in ADRP-CFP-expressing cells electroporated with the indicated viral transcript (WT, wild type). Panels show ADRP, core, and merged images. The D88 mutant showed enhanced accumulation of core around LDs. For all micrographs, scale bars represent 10 µm.

To investigate core trafficking, we performed ReAsH labeling and live cell imaging of core(TC) in Huh-7.5/GFP-ADRP cells. Across multiple experiments, core consistently localized to: i) a dim reticular pattern, most likely the ER (Figure S3E) [13], [22]; ii) caps on ADRP-positive LDs; and iii) bright puncta that were not associated with ADRP-positive LDs (Figure 2A). Notably, the caps of LD-associated core protein frequently faced the ER-like form of core, and core-positive LDs exhibited little directional movement (Figure 2B, upper panel and Video S1). In contrast, ADRP-positive LDs that lacked core were more motile. Furthermore, core puncta were small and motile (Figure 2B, lower panel and Video S1).

Given the velocity of core puncta, we examined whether they were associated with microtubules by performing live cell imaging in Huh-7.5 cells that stably expressed fluorescent protein-tagged ß-tubulin. As shown in Video S2, core puncta trafficked along microtubules, frequently in a retrograde direction. In contrast, motile core puncta were not associated with fluorescent protein-tagged actin filaments (data not shown). Furthermore the motility of core puncta was inhibited by nocodazole, an inhibitor of microtubule trafficking (Figure 2C and Video S3). These data indicated that motile core puncta were trafficked on microtubules.

To better clarify the interaction of core with LDs, and to determine whether core puncta represent small LDs that lack ADRP, we examined additional LD markers. Staining with a dye specific for neutral lipids confirmed that core localized to semi-spherical caps on the surface of LDs and to small LD-independent puncta (Figure S3F). Furthermore, LD-associated core specifically trafficked to ADRP-positive LDs but not to Rab18-positive LDs (Figures S3G and H), which likely represent LDs undergoing lipolysis [33]. These data confirmed that core trafficked to ADRP-positive LDs and to distinct motile puncta that were not LD-associated.

To determine whether the different forms of core corresponded to sites of virus particle assembly, we examined the localization of core with respect to the viral E2 glycoprotein (a structural component of virus particles) or NS3 serine protease-RNA helicase (a NS protein that has been implicated in virus assembly). Since we currently lack tools to image functional E2 and NS3 in live cells, these viral proteins were localized by IF in fixed cells. As seen in Figure 2D, the majority of E2 staining was in a reticular pattern, consistent with its ER retention, as well as discrete E2 puncta that co-localized with core (Figure 2D, arrowheads). These core-E2 structures were frequently found adjacent to small LDs and may represent areas where core has been recruited from LDs into nascent virus particles. The frequency of such core-E2 puncta was low (2.86±0.54 per cell, n = 28 cells), which could reflect the low burst size of HCV as well as the inherent difficulties with antibody recognition of E2 [34]. In addition, we also observed core puncta that were not labeled with E2. Similarly, NS3 also showed a reticular staining pattern, but frequently concentrated in regions adjacent to LD-associated caps of core protein (Figure 2E, arrowheads). For further characterization, we looked for co-localization of core with other components of the secretory compartment, including markers for ER exit sites (Sec16, Sec23), the ER-Golgi intermediate compartment (ERGIC-58), and Golgi (TGN38), but did not reproducibly observe co-localization with these markers (data not shown). Based on these data, we hypothesize that LD-associated core caps likely represent sites of early virus particle assembly, while motile puncta may represent core-containing transport vesicles.

To further clarify the relationship between core trafficking and virus particle assembly, we examined core trafficking in a mutant virus, D88, which is blocked at an early stage of virus particle assembly due to a large in-frame deletion in the E1-E2-p7 genes [35]. The Jc1/core(TC) D88 mutant showed a substantial increase in core accumulation on the surface of LDs (Figures 2F, S3I). While a small number of core puncta were observed, they were non-motile (Video S4), indicating that they were distinct from the motile core puncta seen in virus-producing cells. While it is not yet clear what these non-motile core puncta represent, these data showed that core accumulates on LDs and that motile core puncta were not seen when virus assembly was blocked at an early step.

### Effects of Brefeldin A on core localization

Our previous results suggested that motile core puncta may represent a post LD-form of core. To further investigate this hypothesis, we treated cells with BFA, a fungal metabolite that disrupts ER-Golgi trafficking by inhibiting the activation of ADP ribosylation factor 1 (ARF1) [36]. The timing and dose of BFA treatment were chosen to minimize effects on HCV RNA replication [37]. Consistent with previous findings [38], BFA treatment inhibited the secretion of virus particles as well as a model secreted protein, causing them to accumulate within BFA-treated cells (Figure S4A–B). As ARF1 can regulate lipid homeostasis [39], [40], [41], we also checked whether our conditions affected LD trafficking. Our BFA treatment conditions had only minimal effects on ADRP protein expression, LD number, and LD volume (Figure S4C), indicating that these conditions could be used to study the trafficking of core protein when ARF1 is inhibited.

**Figure 3:**
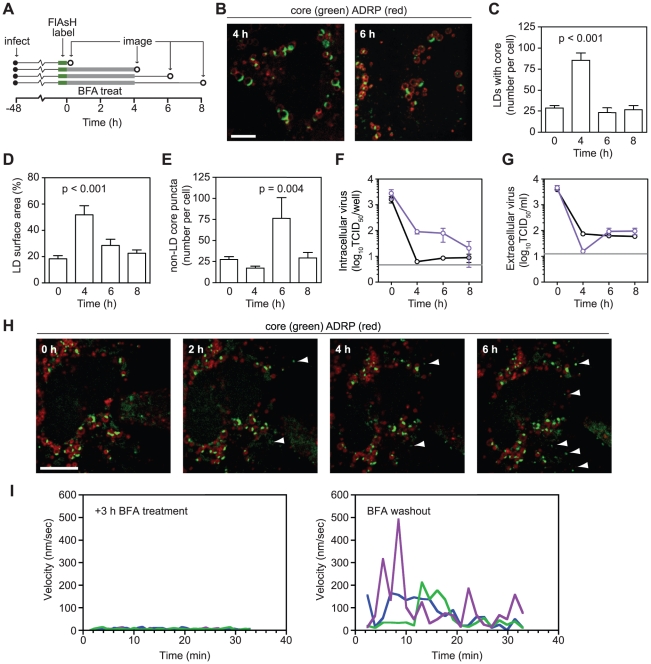
Effects of Brefeldin A on core localization. (A) Schematic of experimental design. Huh-7.5 cells expressing fluorescent protein-tagged ADRP were FlAsH-labeled at 48 h post-infection. Parallel sets of cells were treated with 1 µg/ml BFA for 4 h before drug wash out. Cells were fixed before (0 h) and after (4 h) BFA treatment and also during washout (6 h and 8 h), as indicated by open circles. (B) BFA treatment caused an increase in ADRP-associated core. Images of cells fixed after BFA treatment (4 h) and after washout (6 h). (C–E) Quantitation of images from each time point (n≥19). Image quantification was performed as described in Materials and Methods. All values show mean ± SEM, p values were calculated by using an unpaired Student's t-test. (C) BFA treatment causes accumulation of core-containing LDs. The mean number of core-containing LDs per cell was calculated for each time point. (D) BFA treatment increases the amount of core on each LD. The mean proportion of LD surface area occupied by core (expressed as a percentage) was calculated for each time point. (E) BFA washout increases the number of non-LD core puncta. The mean number of core puncta per cell was calculated for each time point. (F–G) Effects of BFA treatment on infectious HCV production. Triplicate samples of cells (F) or supernatants (G) that had been treated with BFA (1 µg/ml) were collected at the times shown in Figure 3A. Mean virus titers for untreated (black) or BFA-treated (blue) samples are shown for each time point (± SEM). The grey line shows the limit of detection of each assay. (H) Formation of core puncta after BFA washout. Cells expressing tagged-ADRP were treated with BFA (1 µg/ml) for 3 h and labeled with FlAsH. BFA was removed by multiple washes with HBSS immediately prior to imaging. Images were collected every hour from the time BFA was washed out (0 h). Arrowheads show newly formed puncta at each time point. For all micrographs, scale bars represent 10 µm. (I) Core motility during BFA treatment and shortly after drug washout. Three core puncta were randomly chosen from representative time courses (see Videos S5 and S6); their velocities were calculated by using Volocity Quantitation software and plotted over time.

To observe the effects of BFA on core localization, cells were imaged before and after BFA treatment, as well as after drug washout (Figure 3A). BFA treatment increased both the number of LDs containing core and the amount of core that accumulated on each LD (Figure 3B–D), suggesting a defect in core egress from LDs. After drug washout, both of these effects were relieved (Figure 3B–D). BFA treatment resulted in a slight reduction of LD-independent core puncta at 4 h (Figure 3E); however, these puncta were not motile when BFA was present (Video S5 and Figure 3I). Upon BFA washout, more core puncta were observed (Figures 3E, 3H) and core puncta quickly regained their motility (Video S6 and Figure 3I). These effects were mirrored in the intracellular accumulation of virus during BFA treatment and an increase in virus secretion after BFA washout (Figures 3F and 3G). The absence of motile core puncta during BFA treatment and their re-emergence upon washout suggests that they represent a post-LD form of core.

### Long-term dynamics of core trafficking

**Figure 4 ppat-1002302-g004:**
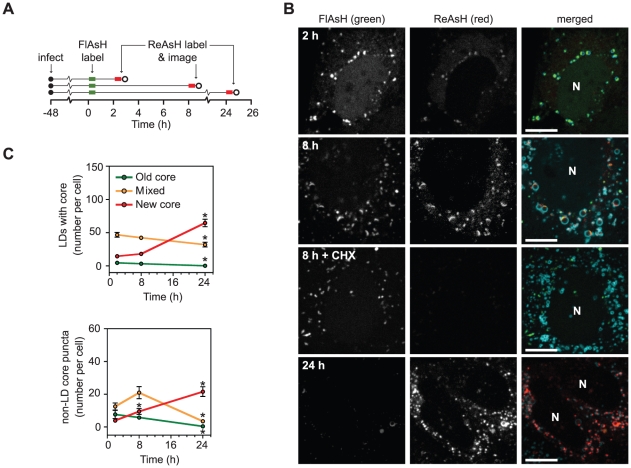
Long term dynamics of core trafficking. (A) Schematic of experimental design. Parallel cultures of Huh-7.5 cells expressing ADRP-CFP were labeled with FlAsH at 48 h post-infection (green bar) then incubated for 2, 8, or 24 h. At the indicated times, cells were labeled with ReAsH (red bar) and imaged (open circle). (B) Localization of newly synthesized core protein over time. Old core labeled with FlAsH (left column) and new core labeled with ReAsH (middle column) are shown together with ADRP in merged images (right column) at each timepoint. For the 8 h timepoint, cells were incubated±30 µM cycloheximide (CHX) to halt protein synthesis. Newly synthesized core was not detected in CHX-treated cells. For all images, scale bar is 10 µm and nuclei (N) are shown for reference. (C) Quantification of old and new core localization over time. The mean number of LD-associated core (top panel) and core puncta (bottom panel) per cell at each timepoint were calculated as described in Materials and Methods. For both graphs, values show mean number of particles at each time point ± SEM (n = 74 to 98). Statistical analysis was used to compare the change in each particle type over time. Asterisks indicate that a statistically significant change was observed in comparison to the previous time point (Student's t-test, p<0.05).

Based on the above results, we expected to observe the trafficking of core from LDs into motile core puncta during live imaging studies. However, these events may be relatively rare, and FlAsH and ReAsH are prone to photobleaching, which greatly limited the number of sequential frames that could be acquired during time course experiments. To better monitor core trafficking over time, we took advantage of the dual labeling capabilities of biarsenical dyes [42]. We reasoned that infected cells could be sequentially labeled under pulse-chase conditions, first with FlAsH to label pre-existing (“old”) core, followed by ReAsH to specifically detect newly synthesized (“new”) core. Pilot experiments showed that simultaneous labeling of Jc1/core(TC)-infected cells with both FlAsH and ReAsH yielded green and red signals that completely overlapped, indicating that both dyes bind TC-tagged core with similar efficiency (data not shown). Next, infected cells were labeled with FlAsH, then labeled with ReAsH after appropriate intervals (Figure 4A). Under these conditions, newly synthesized protein was specifically detected with ReAsH, but not when protein synthesis was halted by cycloheximide treatment during the chase period (Figure 4B, compare second and third rows). Furthermore, newly synthesized core did not traffic to LDs when the maturation of core was blocked by treatment with an inhibitor of signal peptide peptidase but was restored after washout of this inhibitor (Figure S5). These data confirmed that the dual labeling technique could be used to specifically label and image old and new core under pulse-chase conditions.

We used dual labeling to observe the trafficking of core synthesized before and after chase periods of 2, 8, and 24 h (Figure 4B). After 2 h, a small amount of newly synthesized core was detected in an ER-like reticular pattern or co-localized with old core in LD-associated caps (Figure 4B, top row). By 8 h, new core and old core had accumulated on LDs to comparable levels, and mixed puncta (containing both old and new core) were abundant (Figure 4B, second row). By 24 h, new core was the predominant species in both LDs and motile core puncta (Figure 4B, bottom row). Similar results were also obtained when the order of FlAsH- and ReAsH-labeling were switched (data not shown).

In order to observe the trends of core trafficking, we quantitated LDs and motile core puncta that contained old, new, or mixed core protein over time (Figure 4C). These data yielded several interesting results. First, newly synthesized core was targeted to LDs shortly after synthesis. Second, the mixing of old and new core on LDs at 8 h indicated that core-containing LDs maintain communication with the site of core synthesis for extended periods of time (see Discussion). Third, the peak of mixed core in puncta (8 h) was observed after the peak of mixed core on LDs (2 h), further supporting our hypothesis that motile core puncta represent a post-LD form of core. Fourth, the small proportion of motile puncta containing old and mixed core at 24 h suggested that once core leaves LDs, it is either packaged into virus particles for secretion, or turned over.

### The interaction between NS2 and NS3 is essential for recruiting core from LDs

**Figure 5:**
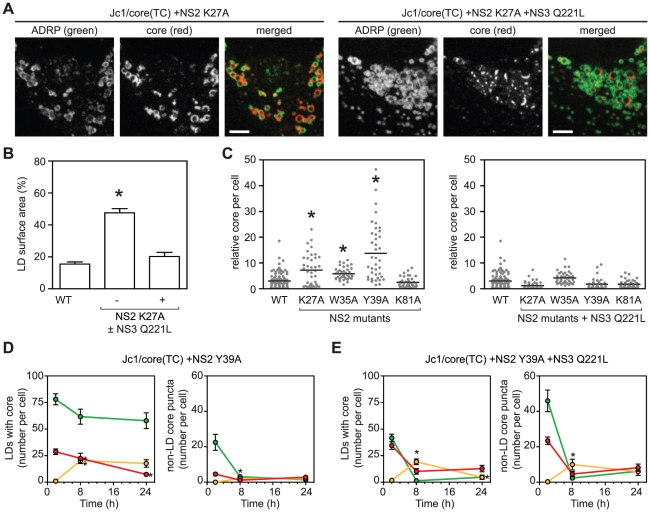
The interactions between NS2 and NS3 are essential for recruiting core from LDs. (A) Localization of core in cells expressing ADRP-CFP electroporated with the indicated viral transcript. Panels show ADRP, core, and merged images; scale bars are 10 µm. (B) The NS2 K27A mutant shows enhanced accumulation of core around LDs. Quantification was performed as described in Materials and Methods; values show mean percent LD surface area occupied by core ± SEM (n≥10). (C) Enhanced accumulation of intracellular core in the presence of NS2 mutations (left panel) ± NS3 Q221L suppressor (right panel). Quantification was performed as described in Materials and Methods (n>38). Horizontal bars represent mean values, asterisks indicate statistically significant differences from WT (Student's t-test, p<0.05). (D–E) Dual labeling of LD-associated core and core puncta in the presence of NS2 Y39A mutation ± NS3 Q221L. Cells were electroporated with the indicated viral transcript then labeled with FlAsH followed by ReAsH after an 8 h chase. The mean number of LD-associated core (left panel) and core puncta (right panel) per cell at each timepoint were calculated as described in Materials and Methods. For all graphs, values show mean number of particles at each time point ± SEM (n = 23 to 71). For comparison with WT, please refer to Figure 4C.

We next examined the role of NS2 in core trafficking. Prior genetic and biochemical studies indicated that NS2 plays an important role in virus assembly by bringing together the viral E1–E2 glycoprotein and NS3-4A enzyme complexes [4], [43], [44], [45]. We previously identified two classes of NS2 mutants with defects in virus assembly [45], [46]. Class 1 mutants (NS2 K27A, W35A, or Y39A) show reduced interaction between NS2 and NS3, and their defects in virus assembly can be suppressed by a second-site mutation in the helicase domain of NS3, Q221L [45], [46]. A class 2 mutant (NS2 K81A) has normal levels of NS2–NS3 interaction but reduced interaction between NS2 and E1–E2, and its defect in virus assembly can be suppressed by a second site mutation in E1, E78T [45], [46]. When the class 1 NS2 mutation K27A was introduced into Jc1/core(TC), the amount of core staining per cell increased, and specifically, core accumulated on LDs (Figures 5A and 5B), similar to what was previously observed with the D88 mutation. The addition of the NS3 Q221L suppressor restored normal core trafficking (Figures 5A and 5B). Based on these results, we tested additional NS2 mutants. All class 1 NS2 mutants showed increased intracellular accumulation of core (Figure 5C, left panel), and specifically, core localized to LDs (Figure S6A). Furthermore, core accumulation was restored to WT levels by the NS3 Q221 suppressor mutation (Figure 5C, right panel). In contrast, the class 2 NS2 mutant, K81A, did not show these effects (Figure 5C). These results were further confirmed by core IF with the untagged Jc1/NS2(K27A) mutant in non-transduced Huh-7.5 cells (Figure S6B). Taken together, these data indicated that the genetic interaction between NS2 and NS3 is important for proper egress of core from the surface of LDs.

To further clarify whether the interaction between NS2 and NS3 is important for the recruitment of core from LDs into core puncta, we performed FlAsH/ReAsH-dual labeling and quantitated LD-associated core and motile core puncta between 48 and 72 h post-electroporation. The NS2 Y39A mutant was used for these experiments because it showed the greatest accumulation of core (Figure 5C). At all time points, the Y39A mutant showed an abundance of LDs containing old core and few LDs containing mixed or new core (Figure 5D, left panel). In contrast, the Y39A + Q221L double mutant showed LDs containing a mixture of old, new, and mixed core (Figure 5E, left panel), which was qualitatively similar to WT (Figure 4C, top panel). Importantly, the number of motile core puncta, especially those containing new core, was significantly reduced in the Y39A mutant (Figure 5D, right panel) but was restored in the Y39A + Q221L double mutant (Figure 5E, right panel). Taken together, we conclude that the interaction between NS2 and NS3 is important for the recruitment of core from LDs into motile core puncta.

## Discussion

We developed methods to image functional core protein in live, virus-producing cells. The small size of the TC tag offered several advantages over fluorescent protein tagging, namely that tagged proteins are more likely to retain native function, and do not require lengthy maturation of protein-encoded fluorophores. Additionally, live cell imaging bypasses the need for fixation, which can alter the localization of LD-associated proteins [47]. In the current study, TC-tagging and labeling with biarsenical dyes had minimal effects on viral replication, core protein expression, or virus particle production. Furthermore, the optimized labeling conditions used here [48] were specific for TC-tagged core protein. Thus, these methods can be used to confidently track functional core protein.

Despite repeated attempts, we did not observe FlAsH-labeled extracellular virus particles. This could be explained by several considerations. First, FlAsH label may be inefficiently incorporated into virus particles, perhaps displaced from the TC tag during RNA packaging. Alternatively, incorporated FlAsH label could be quenched or too dim to image reliably. The detection of extracellular virus particles may also be hampered by the low burst size of HCV [34], the inherent pulse-type labeling with biarsenical dyes, and the propensity of these reagents to photobleach. Nevertheless, we were able to reliably image intracellular forms of HCV core protein.

Three forms of core protein were observed in live cells: ER-associated core, LD-associated core, and motile, LD-independent puncta. On LDs, core formed polarized caps that displaced ADRP and were frequently in close apposition to ER-localized core. Similar LD-associated caps of core protein were previously seen in fixed cells by IF staining [24], [49], and likely reflect sites of core protein transfer between the ER and LDs. Consistent with this model, newly synthesized core was directed to LDs that already contained older core. These data suggest that core-containing LDs maintain direct communication with the ER for extended periods of time or that LDs containing old or new core can fuse to allow mixing.

Large core-containing LDs were immotile during the time periods we observed, which coincided with the peak of virus particle assembly. Prior studies have shown that infection with HCV strain JFH-1 or overexpression of core protein causes microtubule-based trafficking of LDs to the perinuclear region [49], [50]. In our hands, attempts to image core at significantly earlier times were unfruitful due to low levels of core expression and dim staining. Nevertheless, we did observe core-containing LDs clustered in the perinuclear region, suggesting that they had either formed there or moved there prior to staining.

Although LD-associated core has been implicated in virus particle assembly, it has been difficult to demonstrate a direct role for core-LD trafficking in this process. Boulant and colleagues proposed that progressive trafficking of core onto LDs strongly correlates with a rise in infectious virus production [24]. Furthermore, the accumulation of core on LDs inversely correlates with the efficiency of virus production, exemplified by the different localization patterns observed for viruses with high (Jc1) and low (JFH1) infectious titers [22], [26]. Similarly, we observed dramatic increases in the amount of LD-associated core with an HCV deletion mutant that is unable to assemble virus particles.

In addition to LD-associated core, we also observed motile core puncta that were not LD-associated. We propose that motile core puncta represent a form of core relevant for virus assembly based on the following considerations: 1) the formation of motile core puncta was blocked in the D88 mutant, which is unable to assemble virus particles (Figure 2F and Video S4); 2) the formation of motile puncta after BFA washout indicated that they represent a post-LD form of core (Figure 3); and 3) newly synthesized core trafficked to LDs before it trafficked to motile puncta (Figure 4). These data suggest that puncta represent transport vesicles containing virus particles or an intermediate in virus particle assembly. For instance, Lai and colleagues recently showed that core may traffic to a compartment containing early endosomal markers during virus particle secretion [51].

It was interesting that core accumulated on the surface of LDs during BFA treatment (Figure 3B–D), suggesting that ARF1 activity is required for the egress of core from LDs. This would be consistent with a defect in virus assembly, as was seen for the D88 mutant (Figure 2F) and the class 1 NS2 mutants (Figure 5). While BFA inhibited virus secretion, intracellular virus particles still assembled during treatment (Figure 3F–G and S4A–B). One possibility is that virus particles made during BFA treatment may utilize the available ER-associated pool of core; presumably then BFA would cause a defect in virus assembly once this pool is depleted. In addition, BFA may slow the egress of core from LDs and reduce the rate of virus assembly. A full accounting of infectious virus production could be used to discriminate between these possibilities.

**Figure 6:**
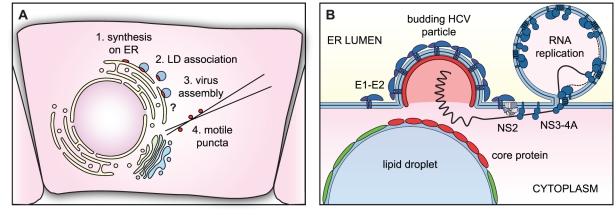
Models of core trafficking and virus assembly. (A) Our model of core protein trafficking is illustrated here. The question mark indicates that the process of virus assembly is still poorly understood. (B) Our model of virus particle assembly. Virus particle assembly likely requires the simultaneous recruitment of core protein from the surface of LDs and viral RNA from replication complexes, while nascent virus particles bud into the ER lumen. NS2 helps to coordinate this process by bringing together viral structural and NS proteins.

Taken together, our data support the model for core trafficking depicted in Figure 6A. Core protein is synthesized on the ER and trafficked to LDs, which remain in direct or indirect communication with the ER. During virus assembly, core protein is recruited from the surface of LDs and into puncta, which likely represent transport vesicles containing virus particles or possibly a core-containing intermediate.

To further explore our model of core trafficking, we utilized our live-cell imaging tools to study the role of NS2 in virus assembly. The class 1 NS2 mutants all showed a large accumulation of core around LDs and fewer core puncta, and core trafficking was restored by the NS3 Q221L second-site suppressor mutation in the viral RNA helicase. In contrast the NS2 K81A mutant showed normal core-LD trafficking. This mutant was previously found to have a distinct defect in virus particle assembly, exhibits normal levels of interaction between NS2 and NS3-4A [45], and is not rescued by the NS3 Q221L mutation [46]. By using sequential labeling, we showed that a block in virus assembly caused an accumulation of old core on LDs and a vast reduction in newly synthesized core on LDs. Thus, the assembly defect results in either reduced synthesis of core, or increased turnover. Importantly, there was a distinct lack of puncta containing newly synthesized core. Taken together, these data strongly suggest that NS2, and more specifically, the interaction between NS2 and NS3-4A, is important to recruit core off the surface of LDs and into core-containing puncta.

Although NS2 is important for the recruitment of core from LDs, NS2 does not directly interact with core protein [43], [44], [45]. How then does NS2 contribute to core trafficking? NS2 coordinates virus particle assembly by bringing together the viral structural and NS proteins (Figure 6B). We propose that the interaction with NS2 may signal to NS3-4A that it is time to stop replicating viral genomes and to start packaging them. Thus, the NS2-NS3 interaction would supply RNAs for packaging; in the absence of proper interaction between NS2 and NS3-4A, core may accumulate on LDs due to a lack of viral RNA for packaging.

In summary, we have developed methods to image functional core protein in live cells. This system allows us to study trafficking of core protein in virus-producing cells, and revealed several novel aspects of core trafficking, including a role for interaction between NS2 and NS3-NS4A in the egress of core from the surface of LDs.

## Materials and Methods

### Plasmids

Plasmids pJc1 and pJc1/Gluc2A were recently described [46]. Jc1/core(TC) was constructed in multiple steps. First, the Jc1 5′ noncoding region (NCR)-core junction was amplified by using KlenTaq-LA (DNA Polymerase Technology, St. Louis, MO) with oligos YO-0021 (5′-AGA CCG TGC ACC ATG AGC TTT CTC AAT TGT TGT CCT GGC TGT TGT ATG GAA CCT AGC GGA TCC ACA AAT CCT AAA CC-3′) and YO-0028 (5′-CCA CGT GCA GCC GAA CCA-3′). The amplicon was cloned into pCR2.1-TOPO (Invitrogen, Carlsbad, CA) and sequenced. The modified 5′ NCR-core junction was then subcloned as a 767-bp ApaLI/AgeI fragment into pJc1 [46] by using common restriction sites.

pJc1/core(TC) D88 was constructed by ligating a 9193-bp ClaI fragment of pJc1/core(TC) and a 1381-bp ClaI fragment from pJc1/NS2(AP) D88 [45]. Plasmids containing NS2 mutations in a Jc1/Gluc2A background were recently described [46]. To facilitate cloning, the Gluc2A gene was inserted into pJc1/core(TC) by using common BsaBI and NotI sites. The EcoRI/MluI fragment from this plasmid was then subcloned into the same sites of Jc1/Gluc2A plasmids containing NS2 mutations, which served to simultaneously introduce the TC tag and excise the Gluc2A gene. Plasmids containing suppressor mutations were created in a similar manner.

To construct lentiviral vectors containing fluorescent protein-tagged cellular markers, pLenti4/EGFP (Invitrogen) was modified to introduce a SpeI site into the multi cloning site. This was done by annealing oligos YO-0183 (5′-GAT CCA CTA GTC TGC AGT CCG GAC-3′) and YO-0184 (5′-CAC TAG TCT GCA GTC CGG ACT CGA-3′) and ligating them into pLenti4/EGFP cut with BamHI and XhoI to create pLenti/MCS. The NheI/Bsp120I fragment from pLA4 (a kind gift of J. McLauchlan [52]) was subsequently cloned into the SpeI/Bsp120I sites of pLenti/MCS to generate pLenti/GFP-ADRP. To create a CFP-tagged version, the NheI/BsrGI fragment of pCerulean-C1 was subcloned into the XbaI/BsrGI sites of pLenti/GFP-ADRP. Fluorescent protein-tagged ADRP was shown to co-localize with anti-ADRP antibody by IF (data not shown). To construct a fluorescent protein-tagged tubulin vector, the tubulin gene was excised from pmTFP-tubulin (Allele Biotechnology, San Diego CA) by using BglII and BamHI and subcloned into the BglII site of pTagRFP-C (Evrogen, Moscow Russia) previously modified to contain a S158T mutation that enhances photostability [53]. The mTagRFP(T)-tubulin gene fusion was then excised with NheI and SacII and subcloned into the SpeI/SacII sites of pLenti/MCS to create pLenti/RFP-tubulin. To express red fluorescent protein-tagged Rab18, mTagRFP(T) was subcloned into pEGFP-Rab18 (a gift from I. Derre) by using common AgeI and BglII sites. A BamHI fragment containing the mTagRFP(T)-Rab18 fusion was then subcloned into pLenti/MCS to create pLenti/RFP-Rab18. For ER-labeling, pmTagRFP(T) was modified to introduce a signal peptide by annealing oligos YO-0412 (5′-CTA GCC GCC ACC ATG GCC CTG CTG AGC GTG CTG CTG CTG CTG GGC CTG CTG GGC CTG GCC GTG GCC AAG-3′) and YO-0413 (5′-GAT CCT TGG CCA CGG CCA GGC CCA GCA GGC CCA GCA GCA GCA GCA CGC TCA GCA GGG CCA TGG TGG CGG-3′). The plasmid was further modified to incorporate an ER retention signal (KDEL) by annealing oligos YO-0426 (5′-GAT CCC GCG CGC CAA GCT TG-3′) and YO-0415 (5′-AAT TCT CGA GTT ACA GCT CGT CCT TCT T-3′) to create pmTag/RFP-KDEL. The signal peptide-RFP-KDEL fusion was then excised by using NheI and XhoI sites and ligated into the SpeI-XhoI sites of pLenti/MCS to create pLenti/RFP-KDEL. pGalT-CFP was a kind gift of N. Altan-Bonnet.

### Cell culture and reagents

Huh-7.5 (a kind gift of C.M. Rice [54]) and 293T cells were maintained in Dulbecco's modified Eagle medium (Invitrogen) containing 10% fetal calf serum (Hyclone, Waltham MA) and 1 mM nonessential amino acids (Invitrogen). Huh-7.5 cell lines expressing fluorescent-tagged proteins were derived by lentiviral transduction. Briefly, lentiviral vectors were packaged in 293T cells by using the ViraPower packaging system (Invitrogen) and used to transduce Huh-7.5 cells. Stable cell lines were selected and maintained by using standard growth medium containing 100 µg/ml Zeocin (InvivoGen, San Diego CA).

Antibodies used for immunoblotting and IF included anti-β actin (Sigma, St. Louis MO), anti-core C7-50 (Affinity BioReagents, Waltham MA), anti-E2 C1 (a kind gift of D. Burton [55]), anti-GFP (Invitrogen) and anti-NS3 (Virogen, Watertown MA). Bodipy 493/503 (Invitrogen) was used to label neutral lipids as per manufacturer's instructions. Transient transfections were performed by using TransIT LT1 (Mirus, Madison WI) or Fugene 6 (Roche, Indianapolis IN) transfection reagents according to manufacturer's instructions.

Nocodazole (Sigma) was dissolved at 25 mM in dimethylsulfoxide and diluted to 50 µM final concentration. (Z-LL)_2_-ketone (EMD BioSciences, Gibbstown NJ) was dissolved at 100 mM in dimethylsulfoxide and diluted to 100 µM final concentration. Cycloheximide (MP Biomedical, Costa Mesa, CA) was dissolved at 100 mM in ethanol and diluted to 30 µM final concentration. Brefeldin A (BFA; Sigma) was dissolved at 5 mg/ml in ethanol and diluted to 1 or 5 µg/ml final concentration. The amount of BFA used and the time of BFA treatment were optimized in pilot experiments by measuring *Gaussia* luciferase secretion (Figure S3A), virus secretion (Figure S3B), and GalT-GFP redistribution (data not shown). Oleic acid (Sigma; 400 ng/well) was added to cells 30 min prior to imaging.

### HCV electroporation and infection

Cells were transfected with HCV RNA transcripts by electroporation, as previously described [56] and seeded onto coverslips in 24-well plates, or into glass-bottom plates (MatTek, Ashland MA). For infection experiments, cells were seeded onto coverslips or glass-bottom plates, and inoculated with virus at 12–24 h post seeding. Luciferase assays were performed on viral supernatants as previously described [46]. Absolute values of viral infectivity were determined by using an endpoint dilution assay as previously described [57]. Briefly, virus was serially diluted into complete growth medium and each dilution was used to infect multiple wells of a 96-well plate. Fifty percent endpoint dilutions were calculated after wells were immunostained with anti-NS5A 9E10 antibody. To measure intracellular infectivity, cells were harvested by trypsinization at 48 h post-transfection, centrifuged at 1200 rpm for 5 min, resuspended in complete medium and subject to three rounds of freezing (−196° in liquid nitrogen) and thawing (37°C). Cellular debris was removed by centrifugation at 10,000 rpm for 5 min and supernatants tested for infectivity as described above.

### Isopycnic ultracentrifugation

Preformed iodixanol gradients were prepared on a Gradient Master 107 (BioComp, Fredericton, Canada) by using 10 and 40% (w/v) Optiprep (Axis-Shield,Oslo, Norway) in PBS. For equilibrium sedimentation, 1ml of each virus was layered on top of the gradient and centrifuged at 40,000 rpm for 14 h at 4°C in an SW-41rotor. Fractions (1ml) were collected from the bottom of the gradient after tube puncture with a fraction collection system (Brandel, Gaithersburg MD). Buoyant densities were determined by refractometry on a handheld refractometer (Reichert, Depew NY).

### RNA quantitation

RNA was extracted from fractions or cell culture medium and quantitated using real-time reverse transcription (RT) PCR as previously described [46]. Briefly, reactions were run with the LightCycler RNA amplification.

HybProbe kit (Roche Applied Sciences, Mannheim, Germany) containing 2 µl RNA sample or RNA quantitation standards, 8 mM MgCl_2_, 375 nM each primer, 250 nM probe, and 1 U RNase inhibitor (United States Biochemical,Cleveland, OH). Reactions were run on a Roche LightCycler 480. Specific infectivity was calculated as the infectivity per RNA copy.

### Tetracysteine labeling with biarsenical dyes

Cells were labeled with FlAsH or ReAsH reagents (Invitrogen) between 48 and 72 h post-infection or post-electroporation with Jc1/core(TC). Immediately prior to diluting, either reagent was mixed with 10 µM 1,2-ethanedithiol (EDT; Sigma). The biarsenical dye-EDT mixture was further diluted in Hanks Balanced Salt Solution (HBBS; Invitrogen) containing 1 mM sodium pyruvate (Invitrogen), 10 mM glucose (Sigma), 1 mM Patent Blue V (Sigma) and 20 µM Disperse Blue 3 (Sigma). Prior to use, Disperse Blue 3 was purified by recrystallization from toluene. After 2 brief washes in HBBS, cells were incubated with FlAsH or ReAsH-containing labeling mixture for 20 min at 37°C, then washed 3 times at 37°C for 10 min with HBSS containing 1 mM 2,3-dimercapto-1-propanol (British anti-Lewisite [BAL]; Sigma), and further washed with HBSS prior to imaging.

For dual labeling experiments, cells were labeled with 2 µM FlAsH, as described above, for 40 min. Immediately after incubation, cells were briefly rinsed once with HBSS and complete growth medium added to each dish. Cells were incubated for 2 to 24 hours, before being labeled with 0.32 µM of ReAsH, as described above, and washed with HBSS containing BAL.

### Immunofluorescence

Cells seeded on coverslips were fixed with 4% paraformaldehyde (PFA) for 20 min at room temperature (RT), then washed twice in PBS containing 100 mM glycine. Cells were permeabilized and blocked in PBS containing 1% BSA (Sigma) with 0.1% saponin (Sigma). Primary antibodies (diluted in the same solution) were added for 1 to 3 h at RT before being washed off with PBS. Secondary Alexa-Fluor antibodies (488 or 568; Invitrogen) were used to label the primary signal. After 1 h incubation and washing, the coverslips were mounted on slides by using Prolong Antifade Gold reagent (Invitrogen).

### Microscopy

Live cell confocal imaging was performed by using a 60× objective on a Nikon TE2000 PFS-2 microscope (Nikon, Tokyo Japan) equipped with a Perfect Focus system (Nikon), Volocity spinning disc confocal unit (Improvision, Waltham MA), and live cell chamber (37°C, 5% CO_2_; Pathology Devices, Westminster MD). Fixed cell fluorescence was performed by using the same instrument without the live cell chamber or Perfect Focus system. Images were edited by using Volocity (Improvision), ImageJ (NIH), or Photoshop (Adobe, San Jose CA) software. Single particle tracking was performed by using the Volocity Quantitation software package (Improvision).

Quantitation of images was performed using the Measurement function in the Volocity software package. To quantify the total number of core particles, the number of objects with volume >0.5 µm^3^ and pixel intensity in the FlAsH or ReAsH channel above background were counted. Background was calculated for each channel as three times the mean pixel intensity of mock-infected cells. Similar methods were used to calculate total LD numbers. LD-associated core was measured by selecting objects with volume >0.5 µm^3^, and pixel intensities greater than background in both the core and ADRP channels. Total core volume was estimated by summing the volume of core particles within the cell. Total cell volume was estimated by tracing the cell outline and using Volocity volume calculations. Relative core per cell was calculated by dividing total core volume by total cell volume. To calculate the surface area of LDs occupied by core, the surface area of core was determined as the sum of the voxels and expressed as a percentage of the total surface area of the LD. Co-localization was performed in ImageJ by using the Co-localization Finder and PSC Co-localization plugins as previously described [58]. Quantitations performed in Figures 3 through [Fig ppat-1002302-g004] 5 were done in a blinded manner.

### Immunoblotting

Huh-7.5 cells (seeded in 6-well plates) were washed twice with Dulbecco's PBS, lysed in 400 µl sample buffer (50 mM Tris [pH 6.8], 100 mM dithiothreitol, 2% [wt/vol] SDS, 10% [vol/vol] glycerol, 0.1% [wt/vol] bromophenol blue), and homogenized by multiple passes through 22- and 28-gauge needles. Electrophoresis and western blotting were performed as previously described [46].

### Statistical methods

Statistical analyses (unpaired Student's t-test) were performed by using Prism 4 software (GraphPad, La Jolla, CA).

## Supporting Information

Figure S1 **Characterization of tagged and untagged Jc1.** (A) The infectivity profiles of Jc1 (top) and Jc1/core(TC) (bottom) are shown after isopycnic centrifugation in a 10 to 40% iodixanol gradient. Infectivity values are plotted against buoyant density. (B) The specific infectivity of each fraction in panel (A) was calculated as the infectivity per RNA copy (TCID_50_/ml divided by RNA copies/ml) and plotted against the buoyant density.
(EPS)

Figure S2 **Effect of fixation and IF processing on FlAsH staining.** Huh-7.5 cells were seeded onto two adjacent cover slips placed in a single 30-mm plate, and infected with Jc1/core(TC). Forty eight hours later, the cells were stained with FlAsH, and one cover slip was imaged under live imaging conditions; the parallel coverslip was fixed in 4% paraformaldehyde, processed for core IF as in Figure 1G, and imaged under identical settings (120 ms exposure; 3 camera gain; 220 camera sensitivity; 2.59 green laser intensity). (A) Representative images from live cells and cells that were fixed and processed for IF. Scale bars, 10 µm. (B) The number of bright FlAsH-labeled objects (average pixel intensity >5000) were counted from 21 random fields by using Volocity software. This threshold was chosen because it discriminates true FlAsH labeling from background fluorescence. Results are shown as the mean ± SEM; the p-value was calculated by using the unpaired Student's t-test.
(EPS)

Figure S3 **Characterization of LDs.** (A) Schematic of the Jc1/Gluc2A reporter genome [44]. Polyprotein cleavage sites are indicated as in Figure 1A. The star indicates C-terminal autocleavage by the foot-and-mouth disease virus 2A peptide, which was fused downstream of the *Gaussia princeps* luciferase gene [44]. Copyright © American Society for Microbiology, Journal of Virology, volume 83, 2009, pp. 8379–8395 doi:10.1128/JVI.00891-09. (B) Huh-7.5 cells expressing tagged ADRP support HCV particle assembly. The indicated cells were electroporated with the Jc1/Gluc2A reporter genome. The infectivity of secreted virus produced by Huh-7.5 (black line), Huh-7.5/GFP-ADRP (green line), or Huh-7.5/CFP-ADRP (blue line) cells are shown for each time point. Data show mean RLU from triplicate experiments ± SEM. The grey line shows the background level of the assay. The mean HCV titers produced at each time point by Huh-7.5/CFP-ADRP and Huh-7.5/GFP-ADRP cells did not significantly differ from the mean HCV titers produced by Huh-7.5 cells (NS; p >0.05; unpaired Student's t-test). (C) ADRP protein expression. The level of endogenous ADRP in Huh-7.5 cells was compared with cells expressing GFP- or CFP-tagged ADRP. Tagged ADRP (open arrowhead) was not overexpressed relative to endogenous ADRP (closed arrowhead). Actin loading controls are shown for comparison between samples. (D) LD volume in Huh-7.5 cells and CFP-ADRP-expressing cells. The average LD volumes were determined in each cell type, with and without oleic acid (OA) treatment (± SEM; n = 65-89), after staining with Bodipy 493/503. Quantitation was performed as described in Materials and Methods. No statistically significant difference in mean LD volume was observed (p >0.05; unpaired Student's t-test). (E) Co-localization of core with ER. Infected cells expressing an ER-retained RFP were labeled with FlAsH and imaged. When the intensity of FlAsH-labeled core was enhanced, co-localization between both signals was observed. (F) Core localized to the periphery of LDs and to LD-independent puncta. Huh-7.5 cells were labeled with ReAsH and Bodipy 493/503 at 48 h post-infection. (G) Localization of LD markers in Huh-7.5 cells. Cells were transiently transfected with fluorescent protein-tagged Rab18 and ADRP constructs and imaged 48 h post-transfection. (H) Core preferentially localizes to ADRP-positive LDs. Cells transiently transfected with ADRP (left panel) or Rab18 (middle panel) were labeled with ReAsH (red) at 48 h post-infection. Co-localization (right panel) was calculated by using Volocity Quantitation software (n = 20). (I) The D88 mutant had enhanced accumulation of core around LDs. Quantification was performed as described in Materials and Methods; values show mean percent LD surface area occupied by core ± SEM (n≥10). All scale bars, 10 µm.
(EPS)

Figure S4 **Effect of BFA on protein and virus secretion.** (A) Extracellular and intracellular luciferase excretion. At 48 h post-transfection, parallel cultures were treated with BFA (5 µg/ml) for 8 h (blue line) or left untreated (black line). Supernatants and cells lysates were harvested at the indicated times and used to determine the levels of extracellular and intracellular luciferase activity, as indicated. (B) Samples from panel A were used to infect naïve Huh-7.5 cells, followed by extensive washing with PBS. The relative levels of extracellular and intracellular infectivity were quantitated by measuring secreted luciferase production at 72 h post-infection. For all graphs, values show mean RLU from triplicate experiments ± SEM. The solid gray bars on each graph indicate the interval of BFA treatment and the black dashed lines indicate the background RLU in these assays. Asterisks indicate significant differences between mock and BFA treated cells (p<0.05; unpaired Student's t-test). (C) Effect of BFA on LDs. Right panel: ADRP protein levels in Huh-7.5 cells expressing CFP-ADRP treated with BFA (1 µg/ml for 4 h) or carrier (ethanol). Levels of tagged ADRP (open arrowhead) and endogenous ADRP (closed arrowhead) were comparable. The LD number (left graph) and LD volume (right graph) ± BFA treatment (1 µg/ml for 4 h) were determined after labeling with Bodipy 493/503. Mean LD number or LD volume per cell (n>23) ± SEM are shown. Asterisk indicates a significant difference between mock and BFA treated cells (p<0.05; unpaired Student's t-test).
(EPS)

Figure S5 **Specificity of dual labeling for old and new core protein.** Parallel cultures of Huh-7.5 cells expressing CFP-ADRP were FlAsH-labeled at 48 h post-infection (green bar), labeled 8 h later with ReAsH (red bar), then imaged (open circle). Old core labeled with FlAsH (left column) and new core labeled with ReAsH (middle column) are shown together with ADRP in merged images (right column). (A) New core is detected on LDs already containing old core 8 h after translation. Note that this is the same image from Figure 4B for comparison. (B) Maturation of newly synthesized core is required for LD localization. Cells were treated with 100 µM signal peptide peptidase inhibitor (SPPi) to inhibit the maturation of newly synthesized core during the chase phase, then labeled with ReAsH and imaged. Newly synthesized core was not targeted to LDs, but to large aggregates. (C) Cells were treated as described in B, but cells were imaged 4 h after SPPi washout. Newly made core was targeted to LDs. For all images, scale bar is 10 µm.
(EPS)

Figure S6 **Intracellular accumulation of core.** (A) Cells expressing GFP-ADRP were electroporated with the indicated viral transcript and labeled with ReAsH 48 h post-electroporation. Enhanced accumulation of core was observed for all class 1 NS2 mutants (K27A, W35A, Y38A) but not the class 2 mutant (K81A). Scale bar is 10 µm. (B). Quantitation of intracellular core in fixed cells. To validate data obtained in Figure 5C, similar quantitation was done on non-transduced Huh-7.5 cells transfected with untagged wild-type (WT) Jc1, Jc1/NS2 K27A, or Jc1/NS2 K27A + NS3 Q221L. Core was visualized by IF with anti-core antibody. Quantitation was done as described in Materials and Methods (n = 34–43 cells per transfection). Horizontal bars represent mean values, asterisks indicate significant differences from WT (p<0.05; unpaired Student's t-test).
(EPS)

Video S1 **Intracellular localization of core protein.** GFP-ADRP expressing cells (green) were labeled with ReAsH (red) at 48 h post-infection. Three forms of core were observed: i) ER-associated core; ii) core associated with ADRP-positive LDs; (iii) ADRP-independent core. ADRP-independent core puncta show motility. Images were captured every 90 sec, scale bar is 17 µm.
(AVI)

Video S2 **Trafficking of core protein on microtubules.** Cells expressing tubulin-RFP cells (red) were labeled with FlAsH (green). Core puncta were seen trafficking on microtubules. Frames were captured every 1 min, scale bar is 17 µm.
(MOV)

Video S3 **Limited trafficking of core protein on microtubules in the presence of 50** µ**M Nocodazole.** Cells expressing tubulin-RFP (red) were treated with 50 µM Nocodazole and labeled with FlAsH (green) 48 h post infection, as described for Video S2. Puncta have reduced motility compared with non-treated cells (Video S2). Frames were captured every 20 sec, scale bar is 17 µm.
(MOV)

Video S4 **Limited motility of core protein in D88 mutant.** Cells expressing tagged-ADRP (red) were electroporated with Jc1/core(D88) and labeled with FlAsH (green). Core puncta are reduced in number and are non-motile. Frames were captured every 15 sec, scale bar is 4.4 µm.
(MOV)

Video S5 **Core motility during BFA treatment.** Huh-7.5 cells were FlAsH-labeled at 48 h post-infection with Jc1/core(TC), treated with 1 µg/ml BFA for 3 h, and imaged for 29 min. Frames were captured every 47 sec, scale bar is 8 µm.
(MOV)

Video S6 **Core motility after BFA washout.** Cells were treated with BFA as in Video S5, washed twice with Hank's buffered saline, and imaged for 40 min. Frames were captured every 91 sec, scale bar is 8 µm.
(MOV)
